# A New View on Lyme Disease: Rodents Hold the Key to Annual Risk

**DOI:** 10.1371/journal.pbio.0040182

**Published:** 2006-05-09

**Authors:** Liza Gross

Any kid who spent summers playing in the woods knew Mom wouldn't let you back in the house without a head-to-toe search for ticks—vectors for a wide range of pathogens throughout the world's temperate regions. In the United States, Rocky Mountain spotted fever was the main concern until 1975, when Lyme disease was found in Connecticut. Since then, incidence has skyrocketed from 497 cases reported in 1982 (the first year national statistics were collected) to a record 23,763 in 2002.

Lyme disease, like other zoonoses, is transmitted by a vector that picks up the pathogen during a blood meal from a vertebrate host. In the eastern and central United States, the spirochete bacterium *Borrelia burgdorferi* infects blacklegged ticks, *Ixodes scapularis*, which feed on a wide variety of birds, lizards, and mammals, including mice, deer, and humans. Since human risk is a function of the prevalence of infection among vectors, outbreak prevention depends in part on understanding what controls infection rates among the agents of transmission.

In a new study, Richard Ostfeld, Felicia Keesing, and colleagues examined the ecological determinants of Lyme disease over a 13-year period in southeastern New York, a hot zone for the disease. Combining field data with computer simulations, they analyzed trends in interannual variation and found two powerful predictors of entomological risk of Lyme disease in a given year: abundance of tick hosts—white-footed mice and chipmunks—in the previous year and abundance of acorns—which sustain the rodents—two years out. Their findings upset the long-held view that deer and climate are the best indicators of disease risk.

*I. scapularis* larvae hatch in midsummer, and acquire infection after feeding on an infected mouse or other small animal. Larvae detach after several days of feeding, then molt into nymphs and enter a nearly year-long dormant stage. After another round of feeding, nymphs fall off and molt into adults, which prefer the blood of larger mammals. Larvae and nymphs can acquire and transmit infection, but people are most likely to contract Lyme disease from nymphs.

A person's risk of exposure to Lyme disease depends on the population density of infected nymphal ticks, which is a product of the total density of nymphs and the nymphal infection prevalence. (Humans can reduce their personal risk by using repellents and routinely checking for ticks when visiting high-risk areas.) Many studies have examined variations in climate and white-tailed deer population dynamics as determinants of tick abundance and disease risk. But few have investigated the impacts of fluctuations in the abundance of hosts for larval ticks, and none have examined all of these variables—temperature, precipitation, deer, mice, chipmunks, and acorns—simultaneously over such a long period.

From 1991 to 2004, the researchers collected temperature and precipitation data, and estimated the abundance of acorns and animals on six plots of land. From this 13-year dataset, they developed computer models to estimate how each of the 11 variables (including multiple climate and deer indexes) contributed to yearly variations in the density of infected ticks and thus risk of human exposure.

While none of the climate variables influenced nymphal infection prevalence, higher temperatures in the previous year and precipitation patterns in the current year had weak, though unexpected, effects on total density and density of infected nymphs. It's thought that higher temperatures keep tick populations down, but the models showed them increasing both total density and density of infected nymphs. And though tick survival is expected to rise with precipitation, the models found the highest tick numbers at intermediate precipitation levels. These inconsistencies can be explored by incorporating other variables with documented effects into the approach outlined here. Also surprising, the researchers found that even a 3-fold variation in deer numbers had no impact on subsequent nymph abundance.

Density of infected nymphs—the principal determinant of Lyme disease risk—varied significantly from year to year, fueled mostly by large fluctuations in total nymph density, which in turn depended mostly on fluctuations in abundance of acorns, mice, and chipmunks. Interestingly, though chipmunk densities are generally lower than mice, their numbers were the best predictor of total nymph density in the subsequent year, likely reflecting their inferior grooming skills. Overall, the results found that acorns were the best predictor of Lyme disease risk—stemming from their crucial role in supporting white-footed mice, chipmunks, and likely other small animals, which in turn provide large reservoirs for *B. burgdorferi*. Acorns will not be a universal predictor of risk, the researchers acknowledge, since the disease occurs in areas without oaks. But the strength of these findings suggests that the observed link between increased Lyme disease risk and high rodent densities indicates that important food sources—or predators—of the rodent hosts of nymphs will be valuable predictors of disease risk.

## 

**Figure:**
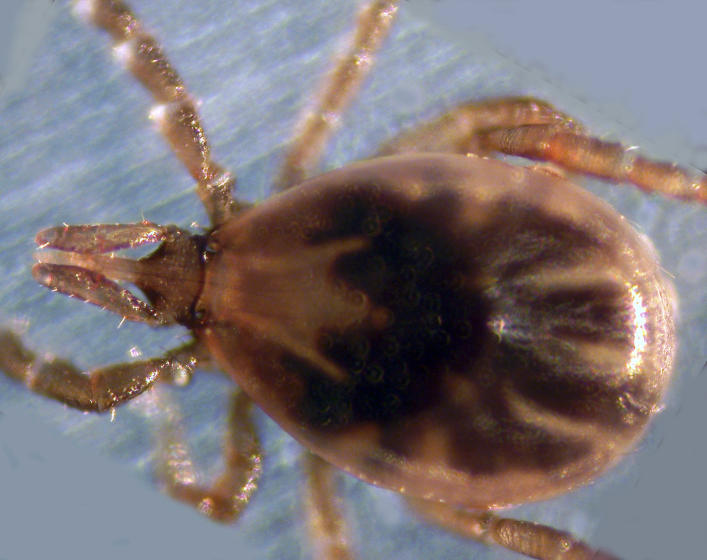
The blacklegged tick ( *Ixodes scapularis*), the primary vector for Lyme disease in the central and eastern United States.

